# Research on Invasive Nontyphoidal *Salmonella* Disease and Developments Towards Better Understanding of Epidemiology, Management, and Control Strategies

**DOI:** 10.1093/cid/ciaa315

**Published:** 2020-07-29

**Authors:** Samuel Kariuki, Ellis Owusu-Dabo

**Affiliations:** 1 Kenya Medical Research Institute, Centre for Microbiology Research, Nairobi, Kenya; 2 Kwame Nkrumah University of Science and Technology (KNUST), School of Public Health, Kumasi, Ghana

**Keywords:** nontyphoidal *Salmonella*, epidemiology, genomics, management

## Abstract

During the 11th International Conference on Typhoid and Other Invasive Salmonelloses held in Hanoi, Vietnam, a number of papers were presented on the burden of disease, epidemiology, genomics, management, and control strategies for invasive nontyphoidal *Salmonella* (iNTS) disease, which is increasingly becoming an important public health threat in low- and middle-income countries, but especially in sub-Saharan Africa (sSA). Although there were minor variations in characteristics of iNTS in different settings (urban vs rural, country to country), it was observed that iNTS has gained greater recognition as a major disease entity in children younger than 5 years. Renewed efforts towards greater understanding of the burden of illness, detection and diagnostic strategies, and management and control of the disease in communities in sSA through the introduction of vaccines will be important.

The 11th International Conference on Typhoid and Other Invasive Salmonelloses invited an increased number of presentations on invasive nontyphoidal *Salmonella* (iNTS) disease with greater coverage in the depth and breadth of data. These included studies covering global burden of disease estimates, understanding the basic and molecular epidemiology of iNTS disease, clinical trials, and efforts towards development and deployment of vaccines for the management of iNTS disease. Presentations clearly demonstrated the advances made in the use of molecular technologies, including whole-genome sequencing (WGS), that provide further insights into our understanding of host–pathogen interactions and the complex evolutionary characteristics of these pathogens. Here, we review the various presentations on iNTS disease made during the conference and make conclusions on advances made and how these affect our understanding of iNTS disease in order to effectively manage the disease, particularly among the most vulnerable populations in sub-Saharan Africa (sSA). Presentations were made on the following themes: global burden of iNTS disease studies; epidemiology and genomics of iNTS disease; management and control of iNTS disease including antimicrobial interventions, antimicrobial resistance, and opportunities for introduction of iNTS vaccines.

## GLOBAL BURDEN OF iNTS DISEASE STUDIES

The availability of burden-of-disease data has been one of the handicaps in our understanding of the clinical importance of iNTS in sSA. In their review of global burden of disease (GBD) by Crump et al entitled, “So is nontyphoidal *Salmonella* invasive disease a neglected disease with many illnesses and deaths?”, it was noted that Ao et al [1] produced the first published estimate of nontyphoidal *Salmonella* (NTS) illnesses and deaths globally for the year 2010. Later the same year, the World Health Organization Foodborne Diseases Epidemiology Reference Group included iNTS disease as a distinct condition with estimates of disability-adjusted life-years (DALYs). And, in 2018, the Institute of Health Metrics and Evaluation based at the University of Washington included iNTS disease as a distinct condition for the first time in the 2017 GBD estimates [2] and established host risk factors for iNTS disease to include infants and young children, malnutrition, recent or current malaria, human immunodeficiency virus (HIV) infection, and sickle-cell disease. Crump et al conclude that, while environmental risk factors have not been well elucidated, iNTS disease is probably associated with unsafe water and poor sanitation and poverty-related conditions and circumstances.

In another analysis by Parisi et al, of the Australia National University, it was noted that, globally, the highest incidence rates for iNTS disease are among children under 5 years of age, with dramatically lower rates among those aged 10 years and older, slight increases around the age of 35 years, and with increasing incidence among those older than 85 years. However, slightly different patterns emerge within different epidemiologic contexts. In high-income settings, the age pattern is U-shaped, with the highest incidence rates among the young and the elderly; in low- and middle-income regions outside of sSA, the highest rates are among children, with lower rates throughout adulthood; while in sSA, the highest rates are among children, but also a pronounced increase in middle adulthood, reflecting the effect of the region’s high HIV prevalence on iNTS risk. The study reported a substantial burden of iNTS at 534 600 cases, 59 100 deaths, and a 14.5% case fatality. At least 24.3% deaths were attributable to HIV, and iNTS disease contributed to 4.3 million DALYs. These iNTS disease parameters are similar to previous estimates from a review by Mahon and Fields [3] and Uche et al [4]. 

## CHALLENGES AND LIMITATIONS WITH GLOBAL BURDEN OF DISEASE ESTIMATES FOR iNTS 

There are very few studies that contribute to disease burden estimates for iNTS, these studies have been conducted in just a few endemic countries, and they are of variable quality. For instance, iNTS disease deaths associated with HIV are assigned to HIV as the underlying cause by the World Health Organization [5] and Murray et al [6]. in their calculations of iNTS burden of disease data. 

## EPIDEMIOLOGY AND GENOMICS OF iNTS DISEASE 

Post et al, from the Institute of Tropical Medicine, Antwerp, reported on iNTS disease transmission studies in Burkina Faso and observed that there was limited overlap between serotypes obtained from human and livestock sources (in contrast to Western countries). In addition, there was a strong genetic association between *Salmonella* Typhimurium isolates obtained from blood culture and stool samples from household members of the patient, with *S.* Typhimurium isolates obtained from human sources living within the same village around the same time. Although the sample size was a limitation, the findings contribute significantly to our knowledge on the role of humans in transmission of pediatric iNTS disease, as previously hypothesized by Kariuki et al [7].

In the same study sites in Burkina Faso, Park et al, from the International Vaccine Institute, observed that *S*. Typhimurium (ST313 and ST19) was the most dominant serovar of iNTS disease in sSA, followed by *Salmonella* Enteritidis (ST11) and *Salmonella* Dublin (ST10). 

These 4 serovars accounted for 87% of iNTS cases detected. All *S*. Typhimurium ST313 belonged to lineage II, with some evidence of transmission between neighboring Ghana and Guinea Bissau. There were 3 lineages associated with *S*. Enteritidis ST11 isolates, and the emergence of a multidrug-resistant (MDR) lineage of *S*. Enteritidis was associated with IncI1 plasmid. Similar findings have been reported by Feasey et al [8] from Malawi. There was a high prevalence of MDR iNTS, with *S*. Typhimurium ST313 exhibiting higher MDR rates than *S*. Enteritidis ST11. Significantly, the study also reported on emerging extended-spectrum B-lactamase–producing *S*. Typhimurium ST313 carrying CTX-M-15 (resistant to ceftriaxone) on both plasmid and the bacterial chromosome. Nonsusceptibility to fluoroquinolones was detected in iNTS isolates from Ghana, similar to previous reports from Kenyan [9] and Democratic Republic of the Congo [10] strains of *S.* Typhimurium. These studies also observed that the current trends suggest a decreasing incidence of iNTS disease in Burkina Faso; however, changes in healthcare utilization in the capital city, Ouagadougou, where the majority of study participants live, may also play a role.

In a study entitled “What have we learned from the 10 000 *Salmonella* genomes project” about the worldwide epidemiology, transmission, and virulence of iNTS by Perez-Sepulveda and colleagues from the University of Liverpool, the team confirmed the dominance of serotypes *S*. Typhimurium in Africa and *S*. Enteritidis as a major cause of iNTS disease in Latin America. ST313 was most common, followed by ST19, and ST11 for *S*. Enteritidis. In the presentation on the Epidemiology of iNTS by Marie-France Phoba from The Democratic Republic of Congo, it was observed that the proportion of intestinal NTS was significantly higher in children admitted with NTS bloodstream infections versus healthy children who were the control (35.1% vs 2.0%). In addition, genomic analysis showed clustering between the majority of paired invasive and intestinal NTS isolates (94.2%). These observations contribute to the understanding of intestinal carriage as a potential source of iNTS infections and add further evidence to the hypothesis of human-to-human transmission of NTS.

## MANAGEMENT AND CONTROL OF iNTS DISEASE

The high rates of antimicrobial-resistant iNTS from endemic areas continue to threaten our ability to effectively treat these infections. A study from Uganda by Winstead et al (Centers for Disease Control and Prevention) evaluating etiologies of bacteremia showed that *Salmonella* was the most commonly detected cause of bacteremia in febrile hospitalized children in Uganda and that 71% of *Salmonella* serotypes causing bacteremia were identified as NTS. Among children with *Salmonella* bacteremia, 61% were diagnosed with septicemia, whereas 45% were given the diagnosis of malaria on admission. Sixty-one percent of children with *Salmonella* bacteremia received ceftriaxone, 44% received gentamicin, and 32% received ampicillin. To help inform these choices, it may be of interest to note that the first-line antibiotics for septicemia in children, as indicated by the 2012 Uganda Clinical Guidelines, are gentamicin plus ceftriaxone, cloxacillin, or benzylpenicillin. Overall, MDR (defined as resistance to ≥2 first-line antibiotics) was seen in 54% of NTS isolates. In this subset, all isolates resistant to nalidixic acid were found to be intermediate resistant to ciprofloxacin. No isolate was resistant to ceftriaxone, azithromycin, or meropenem. As the challenges posed by MDR iNTS increase, the alternative to antimicrobial use for management of iNTS disease will be the development of vaccines, used alone or in combination with effective antimicrobial agents. In a study entitled, “Characterizing the cellular and humoral immune response to invasive nontyphoidal *Salmonella* (iNTS) disease in West African populations” by Sean Elias (Jenner Institute, Oxford), it was observed that antibodies to NTS antigens are nearly universally present in participants studied in Ghana. In addition, serum immunoglobulin (Ig) G and IgA are acquired in parallel from a very young age and are markers of exposure. This serum antibody acquisition appears to also hold true in East African populations [11] and in other concurrent studies in Africa [12–14]. It is also worth noting that iNTS disease drives acquisition of multifunctional CD4+ T cells. Very high titers of NTS antibodies against *Salmonella* LPS may, however, be problematic as they impair immunity against NTS bacteremia, and not just in African adults with HIV as previously shown by MacLennan et al [15].

Baliban et al, from the University of Maryland, reported on the development of a novel trivalent typhoid-iNTS glycoconjugate formulation. They observed that immunization with the trivalent typhoid-iNTS conjugate formulation elicited robust IgG responses to all 3 polysaccharide antigens and anticore and O polysaccharide (COPS) IgG antibodies directed primarily against serogroup-specific O polysaccharide (OPS) epitopes. In addition, postvaccination rabbit sera mediated functional opsonophagocytic activity (*S*. Typhimurium + *S*. Enteritidis) and serum bactericidal activity (*S*. Typhimurium) in vitro. There is need for more immunology studies and the search for more novel vaccine candidates that will provide further insights into the possible use of vaccine for effective prevention and control of iNTS disease.

## CONCLUSIONS AND FUTURE DIRECTIONS

Great strides have been made in the last decade towards our understanding of NTS disease, and especially toward making a distinction between the well-characterized enteric disease and the fairly recently evolved iNTS disease that is predominant in sSA. However, we still have significant gaps in many areas of research on iNTS. First, we require more epidemiologic and genomic data on iNTS from a wider range of countries in sSA to unravel evolutionary trends, source and transmission dynamics of iNTS, and the ecology and disease pathogenesis in endemic settings. Commitments for resources in these settings should be targeted towards accelerating possible inclusion of vaccine introduction. The interaction of iNTS disease with comorbidities such as HIV and malaria needs to be understood. More data on immunologic characteristics of the major iNTS serovars and genotypes will be crucial for informing policy on the development of novel vaccine candidates. Additional resources should also be targeted towards understanding iNTS in countries/regions where we have little or no data on incidence, epidemiology of disease, antimicrobial resistance, and mortality attributable to iNTS. In areas with a high burden of iNTS disease, investment in the promotion of use of currently available treatment options and acceleration of the development of vaccines that could be deployed for the prevention of iNTS disease should be prioritized as a valuable tool for the medium-term and long-term management of this disease.

## Notes

***Acknowledgments.*** All authors of articles reviewed in this manuscript provided written authority for use of their material.

***Financial support.*** S. K. was supported by the National Institute of Allergy and Infectious Diseases of the National Institutes of Health (grant number R01AI099525).

***Supplement sponsorship.*** This supplement is funded with support from the Coalition against Typhoid Secretariat, housed at the Sabin Vaccine Institute in Washington, DC and made possible by a grant from the Bill & Melinda Gates Foundation.

***Potential conflicts of interest.*** The authors: No reported conflicts of interest. Both authors have submitted the ICMJE Form for Disclosure of Potential Conflicts of Interest.
